# Sexual uses of drug and alcohol among men who have sex with men in China: implications for HIV prevention

**DOI:** 10.1186/s12879-022-07880-8

**Published:** 2022-11-29

**Authors:** Jinshen Wang, Peizhen Zhao, Wenqian Xu, Cheng Wang

**Affiliations:** 1 Present Address: Dermatology Hospital, Southern Medical University, 510095 Guangzhou, China; 2 School of Public Health, Southern Medical University, 510515 Guangzhou, China; 3 Southern Medical University Institute for Global Health, 510095 Guangzhou, China

**Keywords:** Alcohol, Drug, Multi-drug use, HIV, Men who have sex with men

## Abstract

**Background:**

Sexual uses of alcohol and drugs are pervasive among men who have sex with men (MSM) and associated with increased risk of HIV infection. However, there are limited studies related to sexual uses of alcohol and drugs among MSM in China. This study aims to describe the pattern of alcohol use, drug use, and multi-drug use during sex among Chinese MSM and to examine the association between condomless anal intercourse, group sex, commercial sex and HIV infection.

**Methods:**

We conducted an online cross-sectional survey in China. Characteristics on social-demographic, sexual behaviors, and sexual uses of alcohol and drugs were collected. The associations with high-risk sexual behaviors and HIV infection were analyzed with multivariable logistic regression.

**Results:**

A total of 699 MSM were included in this study. About 39.5% (230/582) of men reported sexual alcohol use in the past three months and 50.8% (355/699) reported sexual drug use. Of those reporting sexual drug use, around 10.7% (38/355) reported having multi-drug use. Factors associated with both sexual uses of alcohol and drugs included: reporting more male sexual partners (alcohol: adjusted odds ratio [*aOR*] = 1.77; drug: *aOR* = 2.12), reporting condomless anal intercourse in the past three months (alcohol: *aOR* = 2.08; drug: *aOR* = 2.08), having ever engaged in group sex (alcohol: *aOR* = 2.04; drug: *aOR* = 5.22; multi-drug: *aOR* = 3.52) and commercial sex (alcohol: *aOR* = 4.43; drug: *aOR* = 4.22 multi-drug: *aOR* = 5.07). Sexual drug use was also correlated with reported HIV-positive status (drug: *aOR* = 2.53, 95% *CI*:1.31–4.90).

**Conclusion:**

Sexual uses of alcohol and drugs are prevalent among Chinese MSM. Interventions to reduce the sexual use of alcohol and other drugs may be warranted among MSM in China.

## Background

Sexual uses of alcohol and drugs are pervasive among men who have sex with men (MSM) [1, 2]. A global substance use survey reported that 58% of MSM had consumed alcohol and over 20% used drugs during sexual activity [3]. In China, the estimated prevalence of alcohol consumption and drug use among MSM was 13.6% and 28.4%, respectively [2, 4]. Previous studies found that sexual uses of alcohol and drugs were associated with a higher risk of HIV acquisition, which is largely due to the significant association between alcohol and drug use and high-risk sexual behaviors [5–7].

Previous studies reported several potential mechanisms that might explain the association between alcohol/drug use and high-risk sexual behaviors (referring to condomless anal intercourse, group sex, and commercial sex in this study) [8]. First, drug and alcohol use can affect the function of users’ nervous system, including cognitive limit and social disinhibition [9], leading users to focus on significant sensory stimuli and ignore risk information (e.g., the possibility of HIV infection), which facilitates easier reception of high-risk sexual behaviors [10, 11]. Second, certain drugs could affect the cardiovascular system, which leads to smooth muscle relaxation or organ congestion [12]. Therefore, MSM who prefer receptive condomless anal intercourse (CAI) often use drugs to relax the anal sphincter and reduce pain during intercourse [13, 14], while in group sex, drugs are often used to increase stamina to facilitate longer sexual encounters [15, 16]. Finally, from psychosocial and behavioral perspectives, the stigma and discrimination faced by MSM may trigger alienation or lack of attachment to the dominant culture, which in turn may result in behaviors in opposition to dominant norms [17], including alcohol and drug use and being engaged in high-risk sexual behaviors [18–20].

Several studies conducted in the United States (US) and Europe have shown that 9.7–28.8% of MSM who used drugs engaged in multi-drug use [1, 21, 22]. The situation is more common among MSM living with HIV, with a rate of more than 40% [23, 24]. The high association might be due to the fact that multi-drug use has a substantial mental impact on users [25], and can lead to further increases in CAI, group sex, and the number of sexual partners among MSM [4, 25].

However, there are limited studies related to sexual uses of drugs and alcohol among MSM in China, and even fewer studies on multi-drug use. This study aimed to describe the pattern of alcohol use, drug use, and multi-drug use before or during sex among Chinese MSM and to examine their association with high-risk sexual behaviors and HIV infection.

## Methods

### Study design and population

A cross-sectional online survey was conducted among Chinese MSM between 14 July 2018 to 28 July 2018. During this period, participants were able to attend and fill out the survey. The online survey link was disseminated to potential participants through local health departments and community-based organizations with Weibo (a microblogging platform) and WeChat (a messaging app). MSM participated in this study by clicking on the link, which directed them to a survey website hosted by WenJuanXing (Changsha Haoxing Information Technology, China), a professional online survey platform that can provide surveys anonymously in China.

Participants who clicked on the survey link were screened for eligibility. Chinese men were eligible to participate if they were born biologically as a male, more than 16 years old, and ever had anal or oral sex with men during their lifetime. To minimize the risk of multiple participation from the same individual, we used a verification code in the survey. Each phone number or WeChat account can receive a verification code once, and each IP address can only access the survey once.

### Measures

Socio-demographic information included age, marital status, education, income, sexual orientation, and disclosure of sexual orientation to health providers, family, or friends. Sexual behaviors included the number of male sexual partners or female sexual partners in the past three months, consistent condom use in the past three months, group sex with men, and commercial sex. Testing history included HIV testing and syphilis testing history. Consistent condom use was defined as always using condoms when engaged in sex. Commercial sex was defined as MSM who bought or sold sex with men.

Alcohol use was defined as ever consuming alcohol before or during sex in the past three months. Drug use was defined as ever using any of the following drugs before or during sex: rush (amyl nitrites), capsule ‘0’(or 5-Methoxy-Diisopropyltryptamine, foxy), G-point liquid (5-Methoxy-Diisopropyltryptamine), viagra (sildenafil), heroin/morphine/opium, marijuana methamphetamine(or crystal meth, ecstasy) and others. Multi-drug use was defined as the using of three or more different drugs before or during sex [26, 27].

### Data analysis

Descriptive analysis was conducted to describe the socio-demographic characteristics, sexual behaviors, sexual uses of alcohol and drug. Chi-square test was used to assess differences in distributions. Univariate and multivariable logistic regressions were conducted to explore factors associated with alcohol use and drug use. The multivariable models were adjusted for age, legal marital status, educational attainment, annual income, and sexual orientation. Besides, a subgroup analysis was performed to explore the correlates of multi-drug use, restricted to participants who had ever used drugs during sex. *P*-value lower than 0.05 was regarded as statistically significant and all analyses were conducted using R (version 4.1.0).

## Results

In total, the online survey link was clicked on 1814 times and 1036 withdrew from the survey before signing the consent form. Among the remaining 778 participants, 5 men were excluded because they did not consent, 59 participants didn’t meet eligible requirements (13 were females, 2 were less than 16 years old, 44 reported no anal or oral sex with men during their lifetime), and 15 duplicates were excluded. A total of 699 men were completed the survey and included in this study, from 103 cities of 29 provinces in China.

### Sociodemographic and sexual behaviors characteristics

Of the 699 participants, most men were between 16 and 35 years old (90.7%, 634/699), unmarried (84.7%, 592/699), had a college degree or above (78.0%, 545/699), had an annual income of less than 9,000 US dollars (63.1%, 441/699), self-identified as gay (70.0%, 489/699). In terms of sexual behaviors, around half of participants had more than one male sexual partner (42.6%, 298/699) in the past three months, 54.3% (316/582) had condomless anal sex in the past three months. More than half of the participants were willing to disclose their sexual orientation to others (68.0%, 475/699). The proportion of men who engaged in group sex and commercial sex was 24.5% (171/699) and 22.6% (158/699), respectively (Table 1).

**Table 1:** Sociodemographic and sexual behaviors characteristics among MSM in China, 2018 (*N* = 699)

Characteristic	Total, *n* (%)	Drug use, *n* (%)		Alcohol use^a^, *n* (%)	
		No	Yes	No	Yes
Total	699	344 (49.2)	355 (50.8)	352 (60.5)	230 (39.5)
Age (years)					
16–25	318 (45.5)	162 (47.1) ^*^	156 (43.9)	166 (47.2)	85 (37.0)
26–35	316 (45.2)	140 (40.7)	176 (49.6)	154 (43.8)	119 (51.7)
36–45	47 (6.7)	30 (8.7)	17 (4.8)	24 (6.8)	16 (7.0)
> 45	18 (2.6)	12 (3.5)	6 (1.7)	8 (2.3)	10 (4.3)
Legal marital status					
Ever married/engaged	107 (15.3)	43 (12.5)	64 (18.0)	35 (9.9) ^***^	61 (26.5)
Never married	592 (84.7)	301 (87.5)	291 (82.0)	317 (90.1)	169 (73.5)
Highest educational attainment					
High school or below	154 (22.0)	81 (23.5)	73 (20.6)	75 (21.3)	50 (21.7)
Some college	180 (25.8)	95 (27.6)	85 (23.9)	95 (27.0)	43 (18.7)
Bachelor’s degree and above	365 (52.2)	168 (48.8)	197 (55.5)	182 (51.7)	137 (59.6)
Annual income (USD)					
<$5000	208 (29.8)	114 (33.1)	94 (26.5)	103 (29.3)	52 (22.6)
$5001–9000	233 (33.3)	115 (33.4)	118 (33.2)	122 (34.7)	77 (33.5)
$9001–14,000	153 (21.9)	68 (19.8)	85 (23.9)	75 (21.3)	60 (26.1)
>$14,000	105 (15.0)	47 (13.7)	58 (16.3)	52 (14.8)	41 (17.8)
Sexual orientation					
Bisexual	178 (25.5)	82 (23.8)	96 (27.0)	71 (20.2) ^**^	77 (33.5)
Gay	489 (70.0)	242 (70.3)	247 (69.6)	265 (75.3)	147 (63.9)
Other	32 (4.5)	20 (5.8)	12 (3.4)	16 (4.5)	6 (2.6)
Numbers of male sex partners in the past three months					
0	117 (16.7)	68 (19.8) ^***^	49 (13.8)	-	-
1	284 (40.6)	164 (47.7)	120 (33.8)	194 (55.1) ^**^	90 (39.1)
2 ~ 5	282 (40.3)	106 (30.8)	176 (49.6)	150 (42.6)	132 (57.4)
>=6	16 (2.3)	6 (1.7)	10 (2.8)	8 (2.3)	8 (3.5)
Disclosure of sexual orientation to health providers, family, or friends					
Yes	475 (68.0)	235 (68.3)	240 (67.6)	254 (72.2) ^*^	146(63.5)
No	224 (32.0)	109 (31.7)	115 (32.4)	98 (27.8)	84(36.5)
Sexual role in anal intercourse with men^a^					
Insertive	213 (36.6)	109 (39.5)	104 (34.0)	130 (36.9)	83 (36.1)
Receptive	260 (44.7)	116 (42.0)	144 (47.1)	160 (45.5)	100 (43.5)
Both	109 (18.7)	51 (18.5)	58 (19.0)	62 (17.6)	47 (20.4)
Condomless anal intercourse in the past three months^a^					
Yes	316 (54.3)	164 (46.6)	152 (66.1)	122 (44.2)	194 (63.4)
No	266 (45.7)	188 (53.4)	78 (38.9)	154 (55.8)	112 (36.6)
Ever had group sex					
Yes	171 (24.5)	36 (10.5) ^***^	135 (38.0)	70 (19.9) ^***^	85 (36.9)
No	528 (75.5)	308 (89.5)	220 (62.0)	282 (80.1)	145 (63.0)
Ever had commercial sex					
Yes	158 (22.6)	38 (11.0) ^***^	120 (33.8)	48 (13.6) ^***^	98 (42.6)
No	541 (77.4)	306 (89.0)	235 (66.2)	304 (86.4)	132 (57.4)
Ever had HIV testing					
Yes	540 (77.3)	272 (79.1)	268 (75.5)	303 (86.1) ^***^	150 (65.2)
No	159 (22.7)	72 (20.9)	87 (24.5)	49 (13.9)	80 (34.8)
Ever had HIV self-testing^b^					
Yes	406 (58.1)	195 (71.7)	211 (78.7)	227 (74.9)	116 (77.3)
No	134 (19.2)	77 (28.3)	57 (21.3)	76 (25.1)	34 (22.7)
HIV testing result^b^					
Positive	32 (5.9)	13 (4.8) ^**^	19 (7.1)	17 (5.6) ^*^	7 (4.7)
Negative/Other	508 (94.1)	259 (95.2)	249 (92.9)	286 (94.4)	143 (95.3)
Ever had syphilis testing					
Yes	365 (52.2)	171 (49.7)	194 (54.6)	201 (57.1) ^*^	110 (47.8)
No	334 (47.8)	173 (50.3)	161 (45.4)	151 (42.9)	120 (52.2)
Ever had syphilis self-testing					
Yes	174 (47.7)	78 (45.6)	96 (49.5)	95 (47.3)	58 (52.7)
No	191 (52.3)	93 (54.4)	98 (50.5)	106 (52.7)	52 (47.3)
Syphilis testing results in the most recent test^c^					
Positive	44 (12.1)	12 (7.0) ^**^	32 (16.5)	21 (10.4)	18 (16.4)
Negative/Other	321 (87.9)	159 (93.0)	162 (83.5)	180 (89.6)	92 (83.6)

*P < 0.05; **P < 0.01; ***P < 0.001

^a^This analysis was restricted to participants who had one or more sex partners in the last 3 months

^b^This analysis was restricted to participants who had ever tested for HIV

^c^This analysis was restricted to participants who had ever tested for syphilis. –, Not applicable

### Drug use characteristics

Around half of men had ever used drugs before or during sex (50.8%, 355/699), and the most common drugs were rush, viagra, capsule ‘0’, G-point liquid (54.4%, 14.5%, 12.7%, 11.4%). Of those who reported any drug use, most had only used one drug (71.3%, 253/355), and 10.7% (38/355) had used three or more types of drugs (Table 2). Compared with those who reported non-drug use, those who reported any drug use tended to be between 26 and 35 years old (49.6%, 176/355), had more sexual partners (52.4%, 186/355), engaged in group sex (38%, 135/355), and engaged in commercial sex (33.8%, 120/355). Of those who reported any drug use, 7.1% (19/268) and 16.5% (32/194) self-reported HIV-positive and syphilis-positive, respectively (Table 1).

**Table 2:** Types of drug use among MSM who had ever used drugs before or during sex in China, 2018 (*N* = 355)

Variable	*N*	%
Types of drugs		
Rush	271	54.4
Viagra	72	14.5
Capsule “0”	63	12.7
G-point liquid	57	11.4
Marijuana	18	3.6
Heroin/Morphine/Opium	14	2.8
Other	3	0.6
Number of types of drug use		
One	253	71.3
Two	64	18.0
Three	35	9.9
Four	3	0.8

### Alcohol use characteristics

About two-fifth of participants drank alcohol before or during sexual activities in the past three months (39.5%, 230/582). Compared with non-alcohol users, alcohol users tended to be married (26.5%, 61/230), self-identified as bisexual (33.5%, 77/230), had more sexual partners (60.9%, 140/230), were not willing to disclose sexual orientation to others (36.5%, 84/230), engaged in group sex (37.0%, 85/230) and commercial sex (42.6%, 98/230), and tended to be HIV positive (4.7%, 7/150) (Table 1).

### Factors correlated with drug use before or during sexual activities

In the multivariable model adjusted for age, marital status, highest educational attainment, annual income, and sexual orientation, six factors were positively correlated with drug use before or during sexual behaviors: had more male sexual partners (adjusted odds ratio [*aOR*] = 2.12, 95%*CI*: 1.34–3.35) in the past three months, had condomless anal intercourse (*aOR* = 2.19, 95%*CI*: 1.57–3.05) in the past three months, ever participated in group sex (*aOR* = 5.22, 95%*CI*: 3.42–7.95) and commercial sex (*aOR* = 4.22, 95%*CI*: 2.76–6.45), reported a reactive HIV testing result (*aOR* = 2.53, 95%*CI*: 1.31–4.90) and reactive syphilis result in the most recent syphilis testing (*aOR* = 2.65, 95%*CI*: 1.27–5.51) (Table 3).

**Table 3:** Factors associated with drug use or alcohol use among participants enrolled in China, 2018 (*N* = 699)

Characteristic	Drug use (*N =* 699)		Alcohol use (*N* = 582)^a^	
	cOR ( 95% CI)	aOR^b^ (95% CI)	cOR (95% CI)	aOR (95% CI)
Age (years)				
16–25	Ref	Ref	Ref	Ref
26–35	1.31 (0.96–1.78)	1.01 (0.71–1.43)	1.51 (1.06–2.15)	1.07 (0.71–1.61)
36–45	0.58 (0.31–1.11)	0.37 (0.18–0.76)^**^	1.30 (0.66–2.58)	0.69 (0.31–1.52)
> 45	0.52 (0.19–1.42)	0.31 (0.11–0.93)^*^	2.44 (0.93–6.41)	1.29 (0.44–3.79)
Legal marital status				
Ever married/engaged	Ref	Ref	Ref	Ref
Never married	0.65 (0.43–0.99)^*^	0.51 (0.31–0.85)^**^	0.31 (0.19–0.48)^***^	0.34 (0.20–0.58)^***^
Highest educational attainment				
High school or below	Ref	Ref	Ref	Ref
Some college	0.99 (0.65–1.53)	1.00 (0.64–1.57)	0.68 (0.41–1.13)	0.75 (0.44–1.27)
Bachelor’s degree and above	1.30 (0.89–1.89)	1.21 (0.81–1.81)	1.13 (0.74–1.72)	1.14 (0.71–1.81)
Annual income				
<$5000	Ref	Ref	Ref	Ref
$5001–9000	1.24 (0.86–1.81)	1.22 (0.82–1.82)	1.25 (0.81–1.94)	1.09 (0.68–1.75)
$9001–14,000	1.52 (1.00-2.31)	1.36 (0.86–2.16)	1.15 (0.98–2.55)	1.22 (0.72–2.08)
>$14,000	1.50 (0.93–2.40)	1.34 (0.79–2.26)	1.56 (0.92–2.65)	1.08 (0.59–1.96)
Sexual orientation				
Other	Ref	Ref	Ref	Ref
Bisexual	1.95 (0.90–4.23)	1.83 (0.83–4.04)	2.89 (1.07–7.80) ^*^	2.59 (0.92–7.31))
Gay	1.70 (0.81–3.56)	1.82 (0.85–3.87)	1.48 (0.57–3.86)	1.62 (0.59–4.44)
Numbers of male sex partners in the past three months				
0	Ref	Ref	-	-
1	1.02 (0.66–1.57)	0.95 (0.60–1.48)	Ref	Ref
2 ~ 5	2.33 (1.50–3.62) ^**^	2.12 (1.34–3.35) ^**^	1.89 (1.34–2.67) ^***^	1.77 (1.24–2.54) ^**^
>=6	2.00 (0.79–5.06)	2.37 (0.90–6.27)	2.16 (0.90–5.16)	2.19 (0.87–5.49)
Disclosure of sexual orientation to health providers, family, or friends				
Yes	Ref	Ref	Ref	Ref
No	1.03 (0.75–1.42)	0.99 (0.71–1.38)	1.49 (1.05–2.13) ^*^	1.22 (0.83–1.79)
Sexual role in anal intercourse with men^a^				
Insertive	Ref	Ref	Ref	Ref
Receptive	1.19 (0.75–1.89)	1.39 (0.95–2.03)	1.19 (0.74–1.90)	1.03 (0.69–1.52)
Both	1.30 (0.90–1.87)	1.28 (0.79–2.07)	0.98 (0.67–1.42)	1.22 (0.75-2.00)
Condomless anal intercourse in the past three months^a^				
No	Ref	Ref	Ref	Ref
Yes	2.19 (1.57–3.05) ^***^	2.08 (1.48–2.93) ^***^	2.23 (1.58–3.15) ^***^	2.08 (1.45–2.98) ^***^
Ever had group sex				
No	Ref	Ref	Ref	Ref
Yes	5.25 (3.50–7.88) ^***^	5.22 (3.42–7.95) ^***^	2.36 (1.62–3.43) ^***^	2.04 (1.37–3.02) ^**^
Ever had commercial sex				
No	Ref	Ref	Ref	Ref
Yes	4.11 (2.75–6.15) ^***^	4.22 (2.76–6.45) ^***^	4.70 (3.15–7.02) ^***^	4.43 (2.89–6.81) ^***^
Ever had HIV testing				
Yes	Ref	Ref	Ref	Ref
No	1.23 (0.86–1.75)	1.10 (0.76–1.59)	3.30 (2.20–4.95) ^***^	2.75 (1.79–4.22) ^***^
Ever had HIV self-testing^c^				
Yes	Ref	Ref	Ref	Ref
No	0.68 (0.46–1.01)	0.69 (0.46–1.03)	0.88 (0.55–1.39)	0.83 (0.51–1.34)
HIV testing result^c^				
Negative	Ref	Ref	Ref	Ref
Positive	2.77 (1.45–5.27) ^**^	2.53 (1.31–4.90) ^**^	2.30 (1.19–4.46) ^*^	1.90 (0.95–3.78)
Ever had syphilis testing				
Yes	Ref	Ref	Ref	Ref
No	0.80 (0.61–1.10)	0.76 (0.56–1.04)	1.45 (1.04–2.03) ^*^	1.39 (0.98–1.98)
Ever had syphilis self-testing				
Yes	Ref	Ref	Ref	Ref
No	0.86 (0.57–1.29)	0.99 (0.65–1.53)	0.80 (0.50–1.28)	0.82 (0.50–1.35)
Syphilis testing results in the most recent testing^d^				
Negative	Ref	Ref	Ref	Ref
Positive	2.60 (1.30–5.26) ^**^	2.65 (1.27–5.51) ^**^	1.68 (0.85–3.30)	1.45 (0.71–2.96)

* *P* < 0.05; ** *P* < 0.01; *** *P* < 0.001

^a^ This analysis was restricted to participants who had one or more sex partners in the last three months

^b^Multivariate logistic regression adjusted for age, legal marital status, highest educational attainment, annual income, and sexual orientation

^c^ This analysis was restricted to participants who had ever tested for HIV

^d^ This analysis was restricted to participants who had ever tested for syphilis. -, Not applicable

### Factors correlated with alcohol use before or during sexual activities

In the multivariable model adjusted for age, marital status, highest educational attainment, annual income, and sexual orientation, five factors were positively correlated with alcohol use before or during sexual behaviors: had more male sexual partners (*aOR* = 1.77, 95%*CI*: 1.24–2.54) in the past three months, had condomless anal intercourse (*aOR* = 2.08, 95%*CI*: 1.45–2.98) in the past three months, ever participated in group sex (*aOR* = 2.04, 95%*CI*: 1.37–3.02) and commercial sex (*aOR* = 4.43, 95%*CI*: 2.89–6.81), never tested for HIV (*aOR* = 2.75, 95%*CI*: 1.79–4.22) (Table 3).

#### Factors correlated with multi-drug use

In the subgroup analysis of drug use, after being adjusted for age, marital status, highest educational attainment, annual income, and sexual orientation, the multivariable model showed that two factors were positively correlated with multi-drug use: ever participated in group sex (*aOR* = 3.52, 95%*CI*: 1.65–7.48) and commercial sex (*aOR* = 5.07, 95%*CI*: 2.29–11.20) (Table 4).

**Table 4:** Factors associated with multi-drug use among MSM who had ever used drugs before or during sex in China, 2018 (*N* = 355)

Characteristic	Multi-drug *N* = 38 (%)	*cOR (*95% *CI)*	*aOR*^b^ (95% *CI)*
Age (years)			
16–25	18 (47.4)	Ref	Ref
26–35	17 (44.7)	0.82 (0.41–1.65)	0.64 (0.28–1.44)
> 36	3 (7.9)	1.20 (0.32–4.47)	0.87 (0.19–4.03)
Legal marital statue			
Ever married/engaged	10 (26.3)	Ref	Ref
Never married	28 (73.7)	0.57 (0.26–1.24)	0.61 (0.24–1.58)
Highest educational attainment			
High school or below	11 (28.9)	Ref	Ref
Some college	9 (23.7)	0.67 (0.26–1.71)	0.69 (0.26–1.83)
Bachelor’s degree and above	18 (47.4)	0.56 (0.25–1.25)	0.60 (0.73–5.07)
Annual income			
<$5000	8 (21.1)	Ref	Ref
$5001–9000	17 (44.7)	1.85 (0.76–4.49)	1.92 (0.27–3.08)
$9001–14,000	6 (15.8)	0.82 (0.27–2.46)	0.91 (0.51–5.91)
>$14,000	7 (18.4)	1.51 (0.51–4.40)	1.73 (0.16–4.50)
Sexual orientation			
Other	2 (5.3)	Ref	Ref
Bisexual	17 (44.7)	1.09 (0.22–5.43)	0.84 (0.16–4.50)
Gay	19 (50.0)	0.42 (0.09–2.06)	0.35 (0.07–1.82)
Numbers of male sex partners in the past three months			
0	6 (15.8)	Ref	Ref
1	11 (28.9)	0.71 (0.25–2.03)	0.68 (0.22–2.09)
2 ~ 5	19 (50.0)	0.88 (0.33–2.33)	0.87 (0.31–2.47)
>=6	2 (5.3)	1.27 (0.23–7.20)	1.36 (0.22–8.41)
Disclosure sexual of orientation to health providers, family, or friends			
Yes	21 (55.3)	Ref	Ref
No	17 (44.7)	1.84 (0.93–3.64)	1.71 (0.83–3.5)
Sexual role in anal intercourse with men^a^			
Insertive	10 (31.2)	Ref	Ref
Receptive	14 (43.8)	0.99 (0.42–2.33)	1.03 (0.42–2.53)
Both	8 (25.0)	1.47 (0.55–3.97)	1.45 (0.51–4.13)
Condomless anal intercourse in the past three months^a^			
No	7 (21.9)	Ref	Ref
Yes	25 (78.1)	2.21 (0.92–5.29)	2.34 (0.94–5.87)
Ever had group sex			
No	14 (36.8)	Ref	Ref
Yes	24 (63.2)	3.22 (1.6–6.49) ^**^	3.52 (1.65–7.48) ^**^
Ever had commercial sex			
No	14 (36.8)	Ref	Ref
Yes	24 (63.2)	4.01 (1.99–8.10) ^***^	5.07(2.29–11.20) ^**^
Ever had HIV testing			
Yes	24 (63.2)	Ref	Ref
No	14 (36.8)	1.93 (0.95–3.91)	1.87 (0.85–4.09)
Ever had HIV self-testing^c^			
Yes	19 (79.2)	Ref	Ref
No	5 (20.8)	0.98 (0.35–2.75)	0.89 (0.29–2.73)
HIV testing result^c^			
Negative	20 (83.3)	Ref	Ref
Positive	4 (16.7)	1.35 (0.43–4.23)	1.60 (0.45–5.77)
Ever had syphilis testing			
Yes	20 (52.6)	Ref	Ref
No	18 (47.4)	1.08 (0.55–2.11)	1.02 (0.5–2.08)
Ever had syphilis self-testing			
Yes	11 (28.9)	Ref	Ref
No	27 (71.1)	0.79 (0.31-2.00)	0.72 (0.26–2.01)
Syphilis testing results in the most recent testing^d^			
Negative	16 (80.0)	Ref	Ref
Positive	4 (20.0)	1.28 (0.40–4.11)	1.02 (0.27–3.78)

* *P* < 0.05; ** *P* < 0.01; *** *P* < 0.001

^a^This analysis was restricted to participants who had one or more sex partners in the last three months

^b^Multivariate logistic regression adjusted for age, legal marital status, highest educational attainment, annual income, and sexual orientation

^c^This analysis was restricted to participants who had ever tested for HIV

^d^This analysis was restricted to participants who had ever tested for syphilis

## Discussion

Sexual uses of alcohol and drugs can aggravate the worsening HIV epidemic worldwide among MSM [28, 29]. Our findings indicated that sexual uses of alcohol and drugs before or during sex were prevalent among Chinese MSM. This study extends the literature by focusing on alcohol and drug use before or during sex among MSM in China and exploring their association with high-risk sexual behaviors and HIV infection. Findings from this study could provide insights into alcohol and drug harm reduction policies as well as HIV prevention programs.

Our study suggested that sexual uses of drugs are prevalent among Chinese MSM, which is consistent with findings reported in England [30], the US [21], and the Netherlands [22]. The high prevalence among Chinese MSM may be related to low-risk perceptions of substance use [31], function on sexual pleasure enhancement [15, 16], the increasing availability of online recreational drugs [32], and the influence of peer pressure and social pressure [33]. Although many countries have conducted many approaches (including treatment and care of those who reported any drug use, prevention and management of harms, access to controlled drugs, monitoring and evaluation, and behavioral psychology therapy) to address the drug-use problems among MSM [34], the use of drugs before or during sex was still prevalent globally. In China, the government has enacted many policies (such as Narcotic Control Law) and programs (such as methadone maintenance treatment) in response to drug use [35], but these policies and programs mainly target persons who inject drugs, and programs for recreational drugs are limited [36]. Hence, given the high prevalence and serious adverse consequences of recreational drug use, there is a need to explore more effective and comprehensive strategies targeting MSM in China.

Our study also observed a highly sexual alcohol use among MSM in China, which was in line with other studies conducted in China [2] and Russia [37]. This is mainly because of Chinese weak alcohol control measures [38], the increasing availability of alcohol [36], as well as the culture of alcohol drinking among Chinese men [39]. The US public health department recommends assessing alcohol users for drinking through a standardized set of screening questions and providing brief cognitive, behavioral, psychological interventions for harmful drinkers through the healthcare system and community [40]. This recommendation has been implemented by several countries [41], rolling out interventions in sexual health clinics and community settings. Many studies [42–44] have proven that when these interventions were implemented in collaboration with the gay community, it was equally applicable to MSM. However, China has not yet implemented screening measures for alcohol users, and the community-based interventions to reduce harmful alcohol use have not been carried out [45]. It is worthy of consideration for the health department in China to provide alcohol screening for alcohol-using men and collaborate with gay-community to implement interventions for harmful drinkers to reduce alcohol use.

We found that sexual drug use was positively correlated with HIV infection, which is close to that reported in Brazil [46] and the British Isles [47]. This might attribute to the following reasons. First, drug use can increase the likelihood of MSM engaged in high-risk sexual behaviors [5, 7]. Second, the anesthetic nature of some drugs facilitates longer sexual encounters, leading to increased rectal, penile, or vaginal trauma, which increases the risk of HIV infection [48]. The WHO recommends adopting a package of harm reduction interventions to reduce HIV transmission among those who reported sexual drug use, including health interventions (such as pre-exposure prophylaxis; behavioral interventions, HIV testing services, and treatment) and structural interventions (such as supportive policy and funding; addressing stigma; acceptable health services; community empowerment) [49]. Many studies have proven the effectiveness of these above interventions in reducing sexual and substance use behaviors among MSM who reported sexual drug use [42, 50, 51]. In recent years, the Chinese government has outlined a series of pragmatic policies to HIV prevention among those who reported any drug use, including strengthening government leadership, free HIV testing, and treatments, et al [52]. To better address the issue, HIV intervention programs should use more innovative approaches or a comprehensive package of services, like the “behavioral-structural” approach, which combines behavioral intervention, psychological intervention, and biomedical components to curtail these ongoing epidemics [53].

There are several limitations to our study. First, this study recruited participants exclusively through the internet, and therefore caution should be made in generalization to all MSM. Second, all the data were obtained through self-report, which may result in reporting bias. For example, certain drugs such as cannabis and heroin are illegal in China, and some participants may under-report out of fear. Besides, MSM who know they were HIV positive may conceal their HIV status for fear of stigma and discrimination, leading to an underestimation of HIV prevalence. However, the fact that the questionnaires were completed by the participants anonymously may help reduce this bias. Finally, this study was a cross-sectional survey, so it is difficult to make causal inferences about the association of sexual uses of alcohol and drugs with high-risk sexual behaviors and HIV infection.

### Conclusion

In conclusion, our study suggested that sexual uses of alcohol and drugs are prevalent among Chinese MSM, and drug use was positively correlated with HIV infection. Chinese policies and programs in response to this issue are limited, which may compromise the impact of current HIV prevention efforts among MSM. More innovative and comprehensive interventions should be implemented for the MSM to reduce the sexual uses of alcohol and drugs in the future.

## Data Availability

The datasets used in the study are available from the corresponding author on reasonable request.

## Abbreviations

MSM: Men who have sex with men
CAI: Condomless anal intercourse
US: United States
WHO: World Health Organization
